# Eurycomanone induce apoptosis in HepG2 cells via up-regulation of p53

**DOI:** 10.1186/1475-2867-9-16

**Published:** 2009-06-10

**Authors:** Yusmazura Zakaria, Asmah Rahmat, Azimahtol Hawariah Lope Pihie, Noor Rain Abdullah, Peter J Houghton

**Affiliations:** 1 Institute of Biosciences, Universiti Putra Malaysia, 43400 UPM, Serdang, Selangor, Malaysia; 2 School of Biosciences and Biotechnology, Faculty of Sciences and Technology, Universiti Kebangsaan Malaysia, 43600 Bangi, Selangor, Malaysia; 3 Bioassay Unit, Herbal Medicine Research Center, Institute for Medical Research, Jalan Pahang, 50588 Kuala Lumpur, Malaysia; 4 Profesor of Pharmacognosy, Department of Pharmacy, King's College London, Franklin-Wilkins Building, 150 Stamford Street, London SE1 9NH, UK

## Abstract

**Background:**

Eurycomanone is a cytotoxic compound found in Eurycoma longifolia Jack. Previous studies had noted the cytotoxic effect against various cancer cell lines. The aim of this study is to investigate the cytotoxicity against human hepato carcinoma cell in vitro and the mode of action. The cytotoxicity of eurycomanone was evaluated using MTT assay and the mode of cell death was detected by Hoechst 33258 nuclear staining and flow cytometry with Annexin-V/propidium iodide double staining. The protein expression Bax, Bcl-2, p53 and cytochrome C were studied by flow cytometry using a spesific antibody conjugated fluorescent dye to confirm the up-regulation of p53 and Bax in cancer cells.

**Results:**

The findings suggested that eurycomanone was cytotoxic on cancerous liver cell, HepG2 and less toxic on normal cells Chang's liver and WLR-68. Furthermore, various methods proved that apoptosis was the mode of death in eurycomanone-treated HepG2 cells. The characteristics of apoptosis including chromatin condensation, DNA fragmentation and apoptotic bodies were found following eurycomanone treatment. This study also found that apoptotic process triggered by eurycomanone involved the up-regulation of p53 tumor suppressor protein. The up-regulation of p53 was followed by the increasing of pro-apoptotic Bax and decreasing of anti-apoptotic Bcl-2. The increased of cytochrome C levels in cytosol also results in induction of apoptosis.

**Conclusion:**

The data suggest that eurycomanone was cytotoxic on HepG2 cells by inducing apoptosis through the up-regulation of p53 and Bax, and down-regulation of Bcl-2.

## Background

Malignant tumors of the liver can be primary or secondary. Most of them are asymptomatic, with normal liver function. The two commonest malignant primary tumors of the liver are hepatocellular carcinoma (HCC) and cholangiocarcinoma. The former is ten times commoner than cholangiocarcinoma, and is one of the commonest malignant primary neoplasma worldwide [1].

HCC is the fifth commonest neoplasm in the world, and third commonest cause of cancer-related death. More than 500 000 new cases are diagnosed annually worldwide. The incidence ranges from fewer than 10 cases per 100 000 population in North America and Western Europe to 50–150 cases per 100 000 population in parts of Africa and Asia [1]. The incidence of HCC has increased in the last decade, reflecting the higher rates of infection by hepatitis-C virus and improvements in the management of cirrhosis. It is uncommon in the UK and accounts for only 2% of cancers. There are 1500 deaths per year due to HCC in the UK and the incidence is increasing annually [1].

In Malaysia, approximately 1223 primary liver cancer cases were diagnosed in the year 2000. However, it is common for cancer to spread to the liver from the colon, lungs, breasts, or other parts of the body. When this happens, the disease is not liver cancer. The cancer in the liver is a secondary cancer. It is named for the organ or the tissue in which it began. Primary liver cancer or HCC on the other hand, arises from the liver cells itself. HCC is very common in the Asian region. The incidence of HCC increases with age and it is commoner in men than in women [2].

HCC is strongly associated with chronic liver infection or hepatitis, especially Hepatitis B & C viruses. The high incidence of chronic liver infection in Asia is the main cause of HCC in this region. Other important risk factors include liver cirrhosis from excessive alcohol consumption as well as ingestion of aflatoxin, a substance which is found in mouldy nuts and grain. HCC, however, is not hereditary and therefore do not run in the families in absence of the above risk factors [2].

The symptoms of liver cancer are often non-specific. The diagnosis of HCC is usually confirmed by imaging and blood tests. Imaging techniques such as ultrasound and CT scan would usually show either a single or multiple swellings in the liver. To confirm the diagnosis of HCC, a tissue sample of the liver swelling can be taken by inserting a needle through the skin into the liver. The biopsy sample is then examined under the microscope to confirm it is indeed a primary liver cancer. However, a biopsy may not be necessary in all cases, especially when the level of alpha feto-protein is very high [2]. At this time, no one knows its exact causes. However, scientists have found that people with certain risk factors are more likely than others to develop liver cancer.

In the past decade, the role of p53 as apoptotic trigger has been well demonstrated by both *in vit*ro and *in vivo *studies (Yu 2006). Over expression of p53 has been found in many types of human malignancies. There is evidence that supports the existence of a high level of p53 alterations in HCC. At present, many studies of p53 focus on its roles in the pathogenesis and development, diagnosis and treatment, and therapeutic effects and prognosis of HCC [3-8].

Natural products are still major sources of new drug development, for example, between 1981 and 2002, 5% of the 1031 new chemical entities approved as a drug by the US Food and Drug Administration (FDA) were natural products and another 23% were natural-product-derived molecules [9]. The use of herbal medicines as alternative medicines has become increasingly popular in Malaysia and throughout the world. The failure of some conventional medical approaches to produce positive treatment results augments the desire to seek alternative herbal therapies. In addition, herbal medicines are perceived by the public to have lesser side effects as compared to allopathic medicine. Many people are turning back to natural remedies, especially herbal medicine as a solution for maintaining health and for the treatment of disease. In Malaysia, one of the most popular herbs is *Eurycoma longifolia*. *E. longifolia *(Simaroubaceae) is one of the most well known folk medicines for intermittent fever (malaria) in Southeast Asia including Myanmar, Indochina, Thailand, Laos, Cambodia and Malayaia [10-13]. *E. longifolia*, a plant in the family simaroubaceae is a tall slender shrub-tree commonly founds as an understorey in the lowland forests up to 500 m above sea level [11].

In Malaysia, *E. longifolia *is identified locally as 'Tongkat Ali', 'Pasak Bumi' or 'Bedara Pahit' in Indonesia 'Ian-don' in Thailand [11,13,14]. In Vietnam, *E. longifolia *is named 'Cay ba binh' translated as 'a tree which cures hundreds of disease' [15]. The roots of this plant are used as folk medicine for treatment of aches, persistent fever, tertian malaria, sexual insufficiency, dysentery, glandular swelling and as a health supplements [16]. From the roots, several classes of compounds have been identified and they included quassinoids, canthin-6-one alkaloids, β-carboline alkaloids, tirucallane-type triterpenes, squalene derivatives and biphenylneolignans. Some of these constituents were shown to possess cytotoxic, antimalarial, antiulcer, antipyretic and plant growth inhibition activities. The quassinoids 14,15 beta-dihydroxyklaineanone inhibited tumor promoter-induced Epstein-Barr virus activation at an in vitro [17]. Two quassinoids, pasak bumi A (also known as eurycomanone) and pasak bumi B exhibited antiulcer activity [18]. Eurycomanone have shown the great anticancer activities towards the leukemia cells, KB IV and P388. The previous studies also reported that Eurycomanone have a cytotoxic effects on colon cancer, breast cancer, lung cancer, skin cancer (melanoma), fibrosarcoma and lung cancer [19]. Hence, this finding may show that *E. longifolia *have a potential to be a new active drug for cancer suppression.

Previous study of methanolic extract showed induction of apoptosis in human breast cancer MCF-7 cell lines via decreasing of Bcl-2 expression [20-26]. Eurycomanone isolated from *E. longifolia *was also proven to inhibit the growth of MCF-7 cells by triggering apoptosis through down-regulation of the anti-apoptotic protein Bcl-2. It is relatively non-toxic in non-cancerous breast cell lines (MCF-10A)[20]. Eurycomanone also showed the antiproliferative effect in human cervical carcinoma, Hela cells and induces apoptosis through the up-regulation of p53[21].

Strategies to develop chemopreventive drugs for use have been evolving for many years [9,22]. Recently, considerable emphasis has been placed on identifying new cancer chemopreventive compounds capable of inhibiting, retarding or reversing the multistage carcinogenesis. However, the potential use of plants as a source of new drug is still poorly explored. The aim of this study is to isolate and purified Eurycomane, an active compound from the roots of *Eurycoma longifolia *and examine the cytotoxic effect in human hepatoma cells, HepG2 and determine the mode of action for the compound in induces cell death.

## Methods

### Plant materials

The dried roots of *E. longifolia *were provided by Prof Dr Azimahtol Hawariah Lope Pihie (National University of Malaysia, Malaysia).

### Extraction and isolation of the compound

The dried roots (500 g) of *E. longifolia *were extracted with methanol under reflux (60°C, 48 hours) and concentrated using rotary evaporator to give dark brown syrup. The methanol extract was partitioned with diethylether (Et_2_O) saturated with water. The aqueous soluble portion was further partitioned with n-butanol (BuOH) and water (1:1). The BuOH extract was subjected to column chromatography on silica gel column and eluted with ethyl acetate (Et_O_Ac); ethanol (EtOH); water (H_2_O) (100:10:1). Fractions were collected, checked by preparative Thin Layer Chromatoghraphy (TLC) using 20% methanol in chloroform as an eluent to give 6 partially purified fractions (F16–F21) of eurycomanone. Fractions contained eurycomanone were further analyzed by High Performance Liquid Chromatography (HPLC).

### Cell culture

HepG2, HM3KO, CaOV3, Hela, CCD11114sk and Chang's liver cells were obtained from the American Type Culture Collection (ATCC, Manassas, Virginia, USA). Cells were maintained in Dulbecco's modified eagle's medium (DMEM; Invitrogen Co., Carlsbad, California, USA) supplemented with 10% heat-inactivated fetal calf serum (Invitrogen Co.), 100 U/ml penicillin and 100 mg/ml streptomycin (Flowlab, Sydney, Australia) in a humidified atmosphere of 5% CO_2 _in air at 37°C. Cells were kept in the logarithmic growth phase by routine passage every 2–3 days using 0.025% trypsin-EDTA treatment.

### Cell proliferation assay

The antiproliferative activity was evaluated by using the MTT method as previously described, with some modifications [23]. Briefly, cells were seeded 24 hours prior to treatment in a 96-well plate at 5 × 10^4 ^cells/well in order to obtain 70% to 80% confluent cultures. Before addition to the culture medium, the crude extract of *E. longifolia *was dissolved in water and eurycomanone was dissolved in DMSO (Sigma Chemical Co., St. Louis, Missouri, USA) and followed by a 2× serial dilution for 10 points ranged from 0.2 μg/ml to 100 μg/ml. The final concentration of DMSO used in the corresponding wells did not exceed 1% (v/v). This concentration of DMSO does not effects cell viability [18]. Control cultures received the same concentration of solvent alone and tamoxifen (Sigma Chemical Co.) was used as a positive control. The 96-well plate was incubated for 72 hours at 37°C in a humidified atmosphere with 5% CO_2_. At the end of incubation, 50 ul of MTT solution (2 mg/ml MTT in plain culture medium; Sigma Chemical Co.) was added to each well. The plate was then incubated for 4 hours. MTT solution was removed and the purple formazan crystal formed at the bottom of the wells was dissolved with 200 μl DMSO for 20 minutes. The absorbance at 570 nm was read on a spectrophotometric plate reader (Thermo Electron Co., Waltham, Massachusetts, USA). The proportion of surviving cells was calculated as (OD of drug-treated sample – OD of blank)/(OD of control – OD of blank) × 100%. Dose-response curves were constructed to obtain the IC_50 _values. All experimental data were derived from at least 3 independent experiments.

### Hoechst 33258 nuclear staining assay

Nuclear staining with Hoechst 33258 (Sigma Chemical Co.) was performed as described elsewhere [17,18,24]. Briefly, the floating and trypsinized-adherent of treated and non-treated cells were collected and washed with PBS. The cells were then fixed with 4% paraformaldehyde in PBS for 30 minutes at 4°C. After washing, the cells were smeared onto microscope slides followed by incubation in Hoechst 33258 at a final concentration of 30 μg/ml at room temperature for 30 minutes. Nuclear morphology was then examined under a fluorescent microscope (Imaging Source Europe GmbH, Bremen, Germany).

### Cell cycle analysis

HepG2 cells were grown in 25 cm^2 ^culture flasks under a humidified 5% CO_2 _atmosphere at 37°C. The seeding density was 5 × 10^4 ^cells/ml. Flasks were pre-incubated for overnight before the solutions of drugs or control were added to each flask. Vinblastine sufate (2.5 mg/ml) was used as a positive control and negative control received only DMSO. The concentration of eurycomanone used in this experiment was 5 μg/ml. After a period of exposure (24, 48 and 72 hours), cells were harvested by trypsinization with trysin/EDTA solution (Sigma-Aldrich, T4049), collected and fixed in ice-cold 70% ethanol for at least 1 h at 4°C. Cell pellet were resuspended with 0.9 ml 1% Triton X-100 in PBS and 100 μl of 1 mg/ml RNase A (Sigma-Aldrich, R5500). The solution was incubated for 30 minutes at 37°C. Cells were stained with 100 μl of 10 μg/ml PI (Sigma-Aldrich, P4170) solution in staining buffer (1% Triton-X 100 in PBS) and protected from light. Stained cells were incubated at room temperature (RT) for 30 minutes. Samples were then put back in ice and 300 μl of staining buffer and analyzed using a Beckman-Coulter FACS Calibur-500 flow cytometry (FCM).

### Apoptosis measurement by flow cytometry

The number of apoptotic cell death induced by eurycomanone was measured by flow cytometry using the BD Pharmingen™ annexin V-FITC, BD 556420 kit. The treated and untreated HepG2 cells were harvested and washed with cold PBS. The cell pellet was re-suspended in 1× binding buffer at a concentration of 1 × 10^6 ^cells/ml. 100 μl of cell suspension was transferred into FCM tube. 5 μl of Annexin V-FITC and 5 μl of PI were added into the cell suspension, followed by gentle vortexing. The staining samples were incubated for 15 minutes at room temperature (RT) in darkness. An additional 400 μl of 1× binding buffer was added to each tube. Samples were analysed by Beckman-Coulter FACS Calibur-500 flow cytometry within 1 hour. 100 000 events was acquired using green channel FL1 for Annexin V-FITC and the red channel FL3 for PI.

### Detection of proteins involve in apoptosis (Bcl-2; Bax; p53; Cytochrome C)

Cells were cultured in 25 cm^2 ^culture flasks (5% CO_2_, 37°C) at seeding density of 5 × 10^4 ^cells/ml. Plates were pre-incubated overnight before the solutions of drug were added to each well. After a period of exposure (24, 48 and 72 hours), cells were harvested by trypsinization with trypsin/EDTA solution and centrifuged at 1800 rpm. Cells were washed twice with 1× PBS and then were fixed with ice-cold 70% ethanol (≤ 1 hour, 4°C), washed twice with 1× PBS and re-suspended in blocking buffer (2% BSA) for 10 minutes, followed by another washing step. Cells pellet were re-suspended in PBS to a concentration 1 × 10^7 ^cells/ml and 100 μl of cells suspension (1 × 10^6 ^cells/ml) were transferred into each sample tube and 20 μl of FITC/PE conjugated antibody were added into tubes followed by gently mixed. The antibodies used in this experiment are: FITC-conjugated Bcl-2 antibody (BD Pharmingen); PE-conjugated Bax antibody (Santa Cruz Biotechnology, Inc.); PE-conjugated p53 antibody (BD Pharmingen); FITC-conjugated Cytochrome C antibody (Santa Cruz Biotechnology, Inc.). The tubes were incubated at RT for 20 – 30 minutes in the dark. The pellets were washed with 2 ml of 1× PBS and the supernatant were aspirated. Cell pellets were re-suspended in 500 μl of 1× PBS and ready to proceed for FCM analysis (Beckman-Coulter FACS Calibur-500)

### Statistical analysis

All values are expressed as the mean ± S.D. Statistical analyses were evaluated by Student's t-test. Probability values p < 0.05 were considered statistically significant.

## Results

### Effect of eurycomanone on cell viability

The anti-proliferative activity of *E. longifolia *will be assessed from its IC_50 _on various cell lines. The aim of preliminary screening was to select the most effective suppression of cell growth against *E. longifolia *treatment. Because of the limited time and financial support for present study, only the cell line which showed most effective inhibition by the extract was selected for further work.

**Figure 1:**
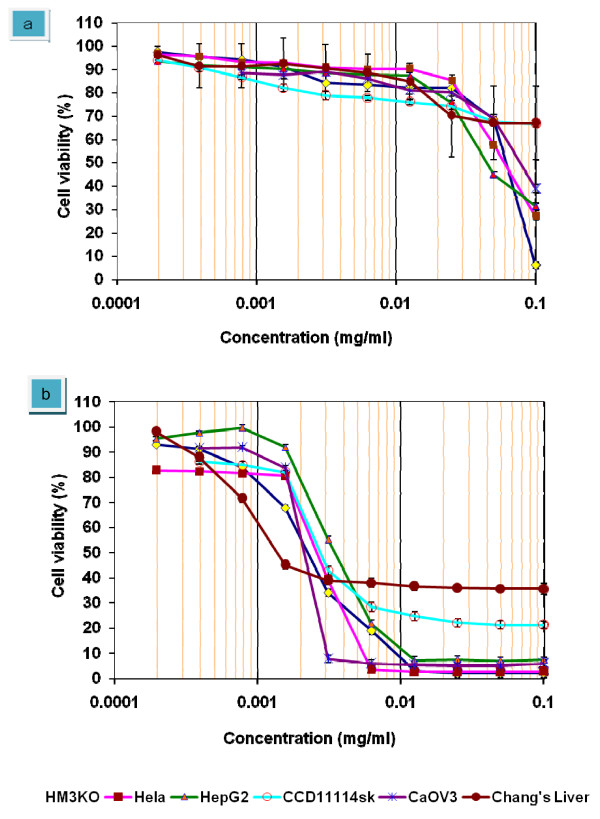
**Dose-response curve of *E. longifolia *crude extract and Tamoxifen against malignant cells (HM3KO, Hela, HepG2 and CaOV3) and non-malignant cells (CCD11114sk and Chang's liver)**. *E. longifolia *crude extract showed a higher toxicity towards HepG2 cells compared to the other cell lines used in the experiment. Non-malignant cells, Chang's Liver and CCD11114sk were not significantly affected by the crude extract and no IC_50 _was evident (a). Tamoxifen showed a cytotoxic effects on all of the cell lines used in the experiment including non-malignant cells, Chang's liver and CCD11114sk (b). The values of graph represent mean ± S.D of three independent experiments. P < 0.05.

Results from the preliminary assay for cytotoxic activity of *E. longifolia *crude extract were evaluated by obtaining the IC_50 _values as in figure 1a. IC_50 _value is an effective dose required to inhibit the proliferative response by 50%. Onset of action and the percentage survival of cells have been taken into consideration in order to categories the potency of the crude extract. In the US NCI plant screening program, a crude extract is generally considered to have *in vitro *cytotoxic activity if the concentration that causes a 50% cell kill (in KB carcinoma cells) is less than 20 μg/ml and less than 4 μg/ml for the pure compound, following incubation between 48–72 h [25]. In this study, this value was used as a rough reference point for assessing the activity of the natural agent used.

Within the four cancer cell lines used in this experiment, crude extract of *E. longifolia *was more effective against HepG2 cells with IC_50 _45 ± 0.15 μg/ml. Both HM3KO and Hela cells gave an IC_50 _value of 60 ± 0.25 μg/ml. For the CaOV3 cells, the IC_50 _obtained was 79 ± 0.16 μg/ml. Tamoxifen, a standard therapeutic drug used as a positive control in this experiment showed a great toxicity towards all the cancer cell lines used. Tamoxifen have been studied intensely particularly on the breast cancer and it was approved by FDA for reducing the incidence of breast cancer. As we can see from the graph in figure 1b, tamoxifen show very low IC_50 _values with 3.7 ± 0.28 towards HepG2 cells and 2.3 ± 0.23 towards HM3KO cells. The IC_50 _values for Hela cells and CaOV3 cells were 2.7 ± 0.18 and 2.1 ± 0.32 respectively.

However, when compared to *E. longifolia *crude extract, tamoxifen not only toxic against malignant cells but also toxic towards non-malignant cell, Chang's liver and CCD1114sk. Tamoxifen gave an IC_50 _value of 2.8 ± 0.21 against CCD11114sk and 1.4 ± 0.31 towards Chang's liver cells displaying the somewhat non-selective cytotoxicity of tamoxifen. In contrast to tamoxifen, no anti-proliferative effect observed in both non-malignant cells. No IC_50 _was recorded.

**Figure 2:**
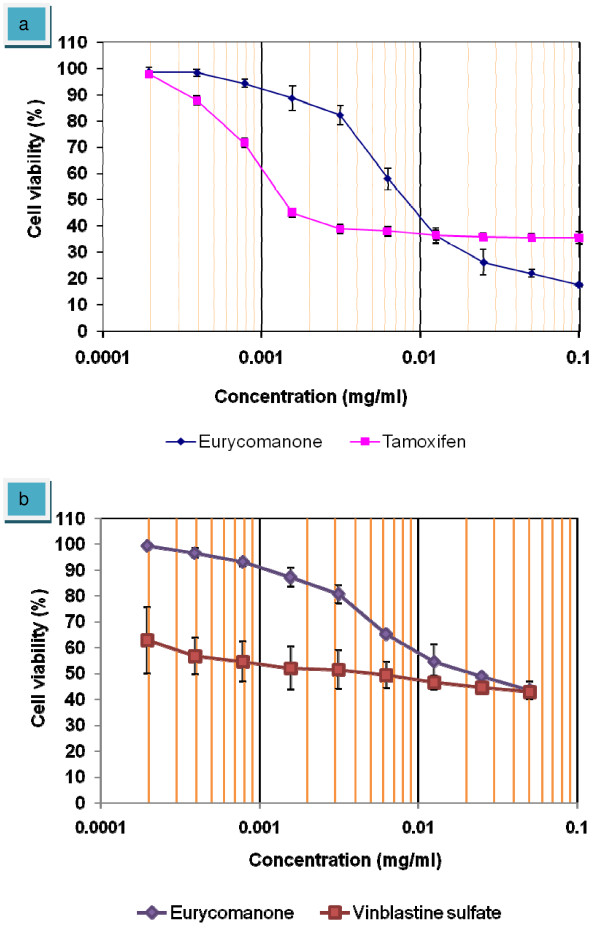
**Dose-response curve of eurycomanone, tamoxifen and vinblastine sulfate against HepG2 cells and WLR-68**. Eurycomanone and tamoxifen signifcantly shown a reduced the number of viable malignant HepG2 cells and non-malignant Chang's Liver cells in a dose dependent manner (a). Eurycomanone and vinblastine sulfate also signifcantly shown a reduced the number of viable non-malignant WLR-68 cells in a dose dependent manner (b). The values of graph represent mean ± S.D of three independent experiments. P < 0.05.

The antiproliferative assay showed a great inhibitory effect of eurycomanone towards HepG2 cells. Eurycomanone as shown in figure 2a, significantly reduced the viability of HepG2 cells by 50% inhibition at 3.8 ± 0.12 μg/ml. When compared to control drug, tamoxifen which is a standard chemotherapeutic drug, the values of IC_50 _for both (eurycomanone and tamoxifen) were very close together. Tamoxifen inhibited HepG2 cells at IC_50 _value of 3.7 ± 0.28 μg/ml. Eurycomanone treatment did not exhibit any cytoselectivity towards normal liver cells, Chang's Liver which gave IC_50 _of 17 ± 0.15 μg/ml. The non-cytoselectivity effect was similar to the treatment with Tamoxifen. However, Tamoxifen is more cytotoxic towards normal liver cells which inhibit 50% cells viability at 1.4 ± 0.31 μg/ml. As compared to Tamoxifen, Eurycomanone was 12 times less toxic against normal liver cells.

Another non-malignant human liver cell, WLR-68 also used in this experiment as a negative control. Interestingly, the results showed that eurycomanone less toxic towards WLR-60 cells with IC_50 _20 ± 0.22 μg/ml (figure 2b). As compared to vinblastine sulfate, this compound showed a highly toxicity towards, normal liver cell WLR-68 with IC_50 _of 4.2 ± 0.37 μg/ml was detected. This finding also supported that eurycomanone have less cytotoxic effect, which mean that the compound highly toxic toward malignant cells but not in normal cells.

### Morphology of apoptosis by Hoechst staining

The mode of killing induced by most anticancer agents is by which apoptotic cell death and DNA fragmentation is a hallmark of apoptotic cells [26]. To further examined the morphological changes in responds to eurycomanone treatment, both control and eurycomanone-treated cells were stained with fluorescent dye Hoechst 33528 and visualized under a fluorescent microscope. Hoechst nuclear staining binds to the AT rich regions of double stranded DNA and exhibits enhanced fluorescence.

**Figure 3:**
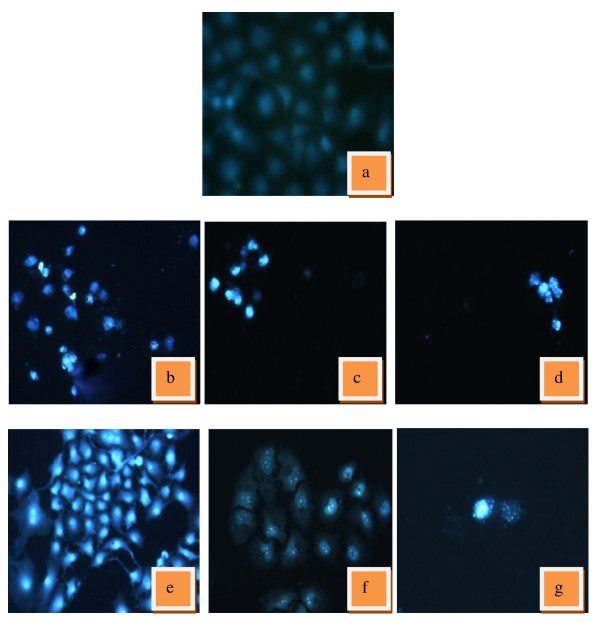
**Nuclear staining of HepG2 cells with Hoecsht 33258**. Cells were treated with eurycomanone and control drug, tamoxifen. (a) Untreated (control) HepG2 cells remained uniformly stained with round and unpunctuated nucleus. (b) Tamoxifen-treated HepG2 at 24 hours. (c) Tamoxifen-treated HepG2 at 48 hours. (d) Tamoxifen-treated HepG2 at 72 hours. (e) Eurycomanone-treated HepG2 at 24 hours. (f) Eurycomanone-treated HepG2 at 48 hours. (g) Eurycomanone-treated HepG2 at 72 hours. Eurycomanone-treated HepG2 cells showed apoptotic morphology; cell shrinkage, DNA condensation and nuclear fragmentation as for tamoxifen.

The Hoechst dye was able to diffuse through intact membranes of HepG2 cells and stain their DNA. Figure 3 showed the results of Hoechst staining for the eurycomanone treated-HepG2 at 24, 48 and 72 hours. The untreated HepG2 cells remained uniformly stained. HepG2 cells show that no fluorescence was detected in the nucleus, as the cells were not apoptotic and did not exhibit DNA fragmentation. At 24 hours treatment, cells began to display apoptotic morphology. Fluorescence was detected in the nuclear region of the HepG2 cells, indicating the presence of DNA fragmentation. Enucleation and apoptotic bodies can be seen as small fluorescent masses. At 48 hours, nuclear condensation and fragmentation was more apparent. The chromatin is condensed into lumps, exhibiting punctuated morphology typical of apoptotic cells, whereas at 72 hours, apoptotic events culminated in the formation of apoptotic bodies, seen as smaller intense spots. Shrinkage of cells, DNA condensations, nuclear and plasma membrane convulsion and nuclear fragmentation were all observed in treated cells. Positive control of tamoxifen-treated cells displayed similar nuclear fluorescence indicating that the data obtained can be accepted. These observations provide evidence that an apoptotic pathway is occurring with the eurycomanone treatment.

### Eurycomanone-arrested cell cycle at G2/M phase

The antiproliferative activity displayed by eurycomanone may be by a selective cytotoxic manner. A wide variety of agents trigger growth arrest as well as cell death [27]. Many reports indicate that cell cycle arrest leads to cell growth inhibition or apoptosis. Cell cycle arrest is a good marker for chemopreventive or anti-tumor activity of chemicals or drugs [28]. However, no reports to show a checkpoint for the cell cycle arrest towards HepG2-treated eurycomanone. Therefore, further investigated the natures of cell cycle progression in HepG2 cells under eurycomanone treatment using a flow cytometer approach were done. The cell cycle distribution was examined at various times and indicated doses. Vinblastine sulfate was used as a positive control.

**Figure 4:**
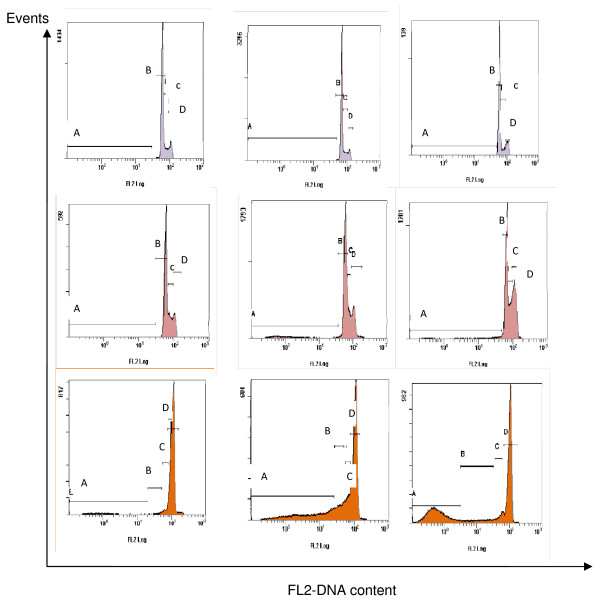
**DNA content of HepG2 cells detected by flow cytometry**. Untreated HepG2 cells (purple square); HepG2-treated eurycomanone (red square) and HepG2-treated vinblastine sulfate (orange square); for 24, 48 and 72 hours (a). A = G0 phase; B = G1 phase; C = S phase; D = G2/M phase. Untreated cells showed the highest peak in the region of G1 phase and a small low peak in G2/M phase. HepG2 cells treated with eurycomanone increased at G2/M peak suggested that, eurycomanone arrested HepG2 cell cycle at G2/M phase. Following vinblastine treatment, as a control drug, the G2/M peak dramatically increased. The significant accumulation of cells clearly indicates that vinblastine sulfate efficiently arrested the cell cycle progress in HepG2 cells at the G2/M phase.

**Figure 5:**
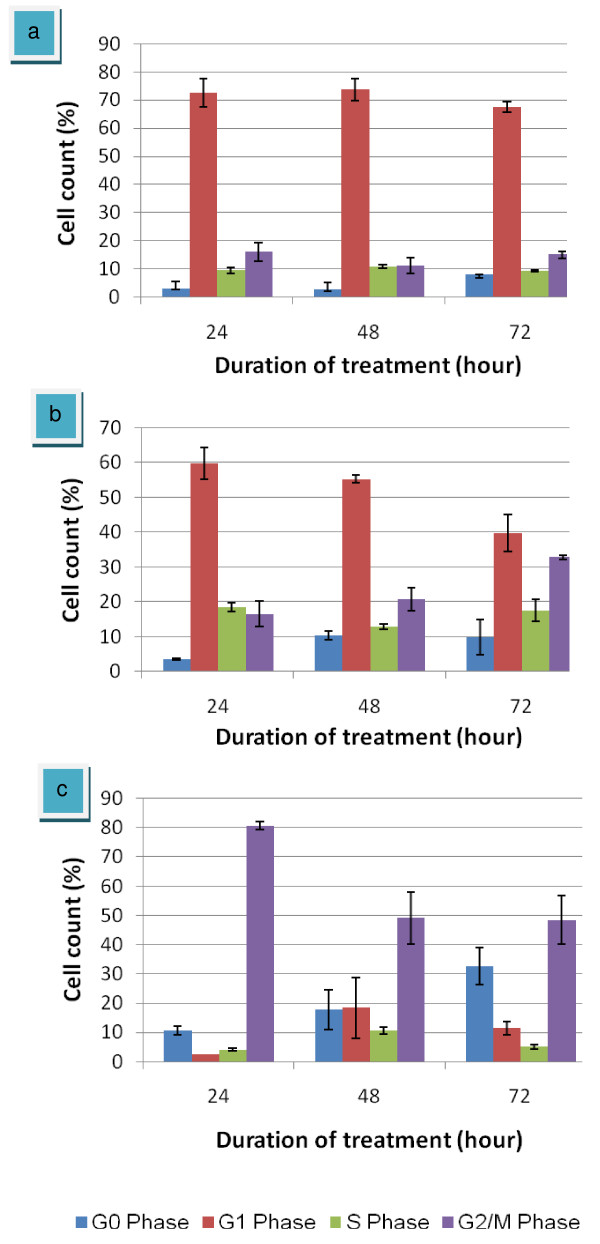
**Graph summarized the DNA contents of HepG2 cells for the various treatments**. Untreated HepG2 cells (B); HepG2-treated eurycomanone (b) and HepG2-treated vinblastine sulfate (c). The values of the bars represented mean ± S.D. of three independent experiments. P < 0.05.

As shown in figure 4, cells under normal conditions (untreated) showed the expected pattern for continuing growing cells by which the highest peak in the region of G1 phase and a small low peak in G2/M phase. The number of cells count was calculated and presented in a graph in figure 5. There were no significant differences within the G1 peak for untreated cells at 24 and 48 hours with percentage cell count of 72.5% and 73.6%, respectively (figure 5a). Besides, at 72 hours of treatment duration, a bit decreased of cells population to 67.5% was observed. Maybe, it is because of the normal process for the cells to go apoptosis following increasing time of incubation. However, most of the cells accumulated at G1 phase.

Cells treated-eurycomanone showed an accumulation in G2/M phase following 48 hours of exposure with 20.8% of the cells population compared to 24 hours with 16.58% (figure 5b). At 72 hours, the significantly increased percentage of cells in G2/M phase was observed with 39.9% of cells accumulated was recorded. Along with decreasing of G2/M phase, a dramatically decreased of HepG2 cells population in G1 phase were detected with percentage of 59.9% at 24 hours to 55.4% at 48 hours and 39.9% at 72 hours. This finding suggested that eurycomanone inhibited the cell proliferation by inducing the G2/M arrest in a time-dependent manner in HepG2 cells through regulating the G2 checkpoint.

The amount of apoptotic cells was calculated based on the appearance of cells in G0. G0 phase is corresponds to apoptotic cells. Although there was an increase of apoptosis in eurycomanone-treated cells, the magnitude of change was relatively small in comparison to the growth inhibition. Distribution of the G2/M phase cells in the population increase while the percent of apoptotic cells rose from 3.5% to 10.4%. This is also evident that the G2/M phase became the dominant phase in cells treated with 5 μg/ml of eurycomanone in a time-dependent manner with a late apoptosis. These finding indicate that at the ranges of concentration studied, the antiproliferative effect of eurycomanone on HepG2 cells could be attributed primarily to the induction of G2/M arrest, with less contribution of cell division rather than DNA synthesis.

Following vinblastine sulfate treatment (figure 5c), which is a control drug, the G2/M peak dramatically decreased cell population of G1 phase (2.5%) and increased percentage of cells in G2/M phase (80.7%) at 24 hours exposure compared to control. Therefore, at 48 h of treatment, G2/M phase decreased to 49.1%, along with increased of G0 phase from 10.6% at 24 hours to 17.7%. Notably, maximal G2/M phase arrest by vinblastine sulfate administration was observed at 24 hours and at 72 hours for eurycomanone. The significant accumulation of cells clearly indicates that vinblastine sulfate efficiently arrested the cell cycle progress in HepG2 cells at the G2/M phase. This result is consistent with those in the literature [29] and testifies that the established cell cycle analysis protocol with HepG2 cell line was working properly in the present work.

### Eurycomanone-induced apoptotic cell death

**Figure 6:**
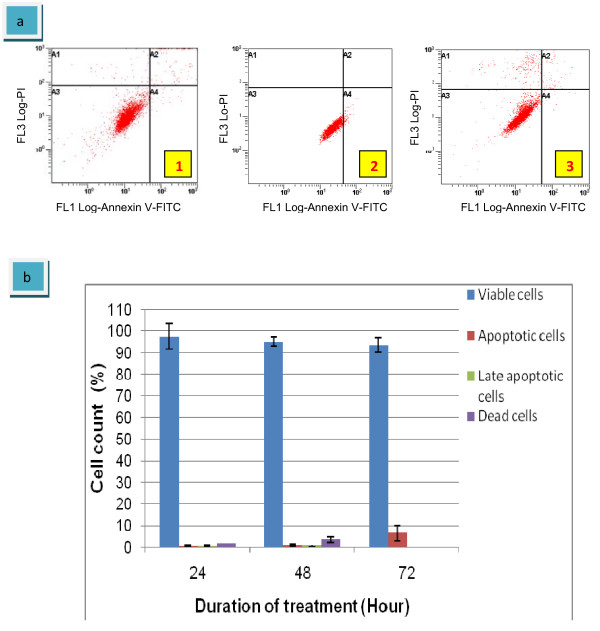
**Scatter plots of Ann/PI-untreated HepG2 cells under four situations in quadrant analysis**. They are living cells (A3), apoptotic cells (A4), late apoptotic or dead cells (A2) and necrotic or dead cells (A1). Exposed for 24 hours (1); exposed for 48 hours (2); exposed for 72 hours (3)(a). The graph summarized the number of cells in each quadrant (b). The values of the bars represented mean ± S.D. of three independent experiments. P < 0.05.

To further confirm that eurycomanone induces cell apoptosis, HepG2 cells were stained with annexin V-FITC and PI, and subsequently analyzed by flow cytometry (FCM). As indicated in figure 6a, in untreated cells only 0.3% of HepG2 cells were annexin V^+^/PI^-^, indicating the apoptotic cells, whereas 0.58% of the cells were double positive (late apoptotic cells) at 24 hours of incubation time. The apoptotic cells showed a bit increased after 48 hours treatment with 0.69% and late apoptotic cells with 0.95%. At 72 hours of treatment duration, only 6.4% of apoptotic cells were detected with 0.04% of late apoptotic cells. Most of the cells for all treatment duration (24, 48 and 72 hours) were still viable with percentage of 97.43%, 95.9% and 93.6%, respectively (figure 6b). This result significantly showed that in untreated HepG2 cells, cells continuously growth and an apoptosis process just appeared as a normal process in every cell lives. Results also supported the finding from the analysis of DNA contents which previously reported in this study.

**Figure 7:**
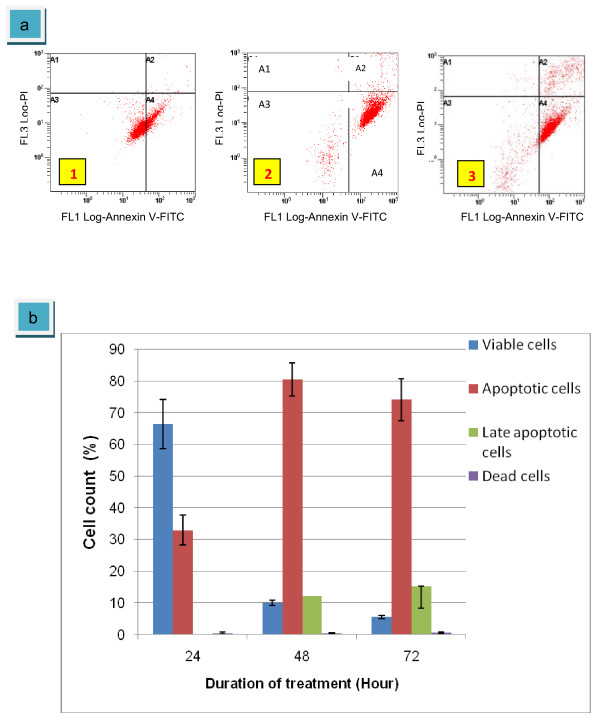
**Scatter plots of Ann/PI-stained HepG2 cells treated-eurycomanone (5 μg/ml) under four situations in quadrant analysis**. They are living cells (A3), apoptotic cells (A4), late apoptotic or dead cells (A2) and necrotic or dead cells (A1). Exposed for 24 hours (1); exposed for 48 hours (2); exposed for 72 hours (3)(a). The graph summarized the numbers of cells in each quadrant (b). The values of the bars represented mean ± S.D. of three independent experiments. P < 0.05.

After treatment with eurycomanone (figure 7a) for 24 hours, apoptotic cells increased to 32.97% along with decreased in viable cells to 65.36%. Following exposure for 48 hours, annexin V^+ ^quadrant dramatically increase to 80.5% and late apoptotic cells also increased with 12.10% were detected. Viable cells decreased to 10.0%. However, at 72 hours of treatment, only 74.1% of the cells were detected in quadrant of annexin V^+^/PI^-^, but the increased of cells population were observed at late apoptotic quadrant with percentage of 15.3%. Eurycomanone was showed to induce apoptosis in HepG2 cells in a time-dependent manner. After 72 hours of exposure, only 5.6% cells were alive indicating that almost all of cells undergo apoptosis (figure 7b). This data confirm that eurycomanone was capable of inducing cell death in HepG2 cells and suggested that the mechanism of cell death in HepG2-treated eurycomanone was via apoptosis process. According to Nurkhasanah et al. 2008, after exposure of Hela cells with eurycomanone, the level of apoptotic cells in annexin ^+^/PI^- ^quadrant was increased at 24 and 48 hours, thus indicating that eurycomanone induced apoptosis in Hela cells. Some of natural resources also reported to induce apoptosis in HepG2 cells such as solanine or *Solanum nigrum *Linn [30] and apigenin [31].

**Figure 8:**
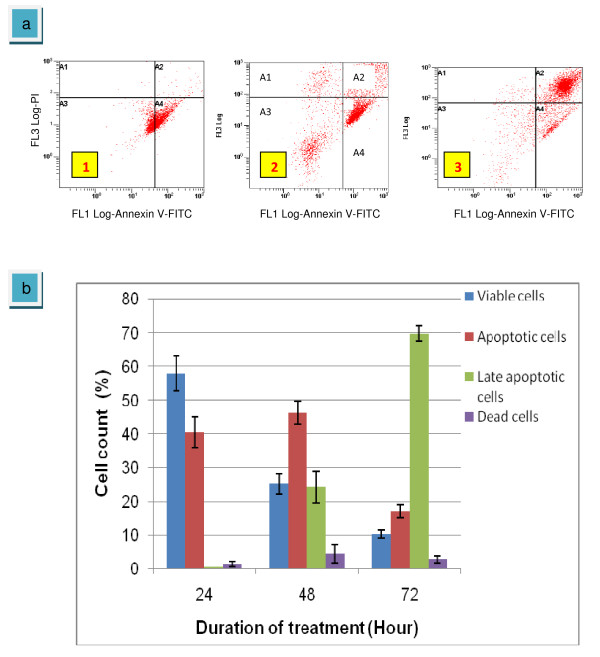
**Scatter plots of Ann/PI-stained HepG2 cells treated-vinblastine sulfate (2.5 μg/ml) under four situations in quadrant analysis**. They are living cells (A3), apoptotic cells (A4), late apoptotic or dead cells (A2) and necrotic or dead cells (A1). Exposed for 24 hours (1); exposed for 48 hours (2); exposed for 72 hours (3)(a). The graph summarized the numbers of cells in each quadrant (b). The values of the bars represented mean ± S.D. of three independent experiments. P < 0.05.

Vinblastine sulfate used as a positive control in this present experiment. Vinblastine sulfate showed a decreasing number of viable cells with increasing time of incubation (figure 8a). At 24 hours, percentage of cells population in double negative quadrant was 57.84% and decreased to 25.1% at 48 h and 10.3% at 72 hours. Interestingly, at 72 hours, late apoptotic cells greatly increased to 69.8% along with 17.0% of apoptotic cells compared to percentage of 46.2% at 48 hours and 40.47% at 72 hours with late apoptotic of 24.2% and 0.38%, respectively (figure 8b). This data have similarity with the results of DNA contents for cell cycle analysis which mentioned above.

### Eurycomanone-induced p53 and Bax up-regulation

To investigate the intracellular mechanism for the observed increase in apoptosis in eurycomanone-treated HepG2 cells, the expression of the pro- apoptotic Bax and anti-apoptotic Bcl-2 proteins will be studied. Also, the involvement of the tumor suppressor protein p53 will be determined along with cytochrome C release. Protein levels discerned from treated and non-treated cells will then be quantitated and compared to observe possible modulation by eurycomanone. Flow cytometry approach was used to detect the levels of proteins with specific antibody conjugated-FITC (fluorescein isothiocyanate) and PE (phycoerythrin).

**Figure 9:**
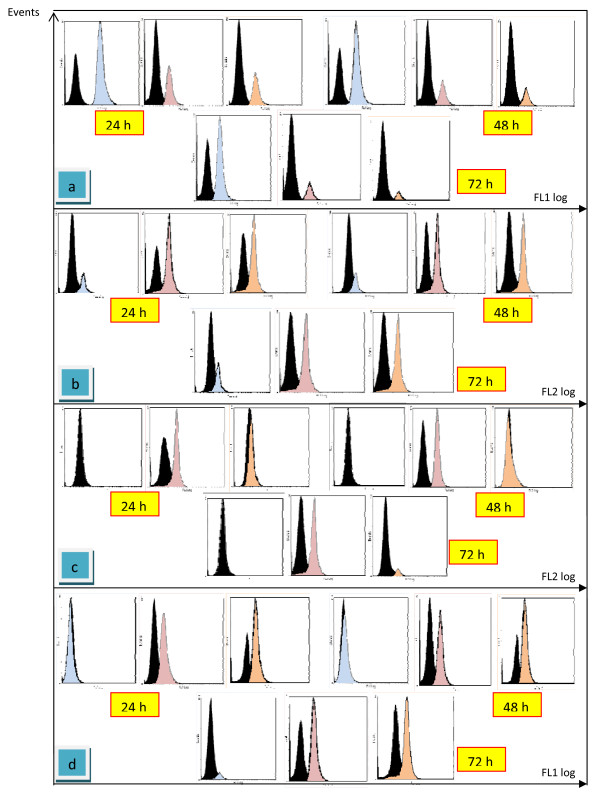
**The histogram of proteins expression detected by flow cytometry with a specific antibody**. The profile of Bcl-2 expression proteins (a); the profile of Bax expression proteins (b); the profile of p53 expression proteins (c); the profile of cytochrome C expression proteins (d), for 24, 48 and 72 hours. (Black square = unstained HepG2 cells; Blue square = untreated HepG2 cells; Red square = HepG2 cells treated-eurycomanone; Yellow square = HepG2 cells treated-vinblastine sulfate).

**Figure 10:**
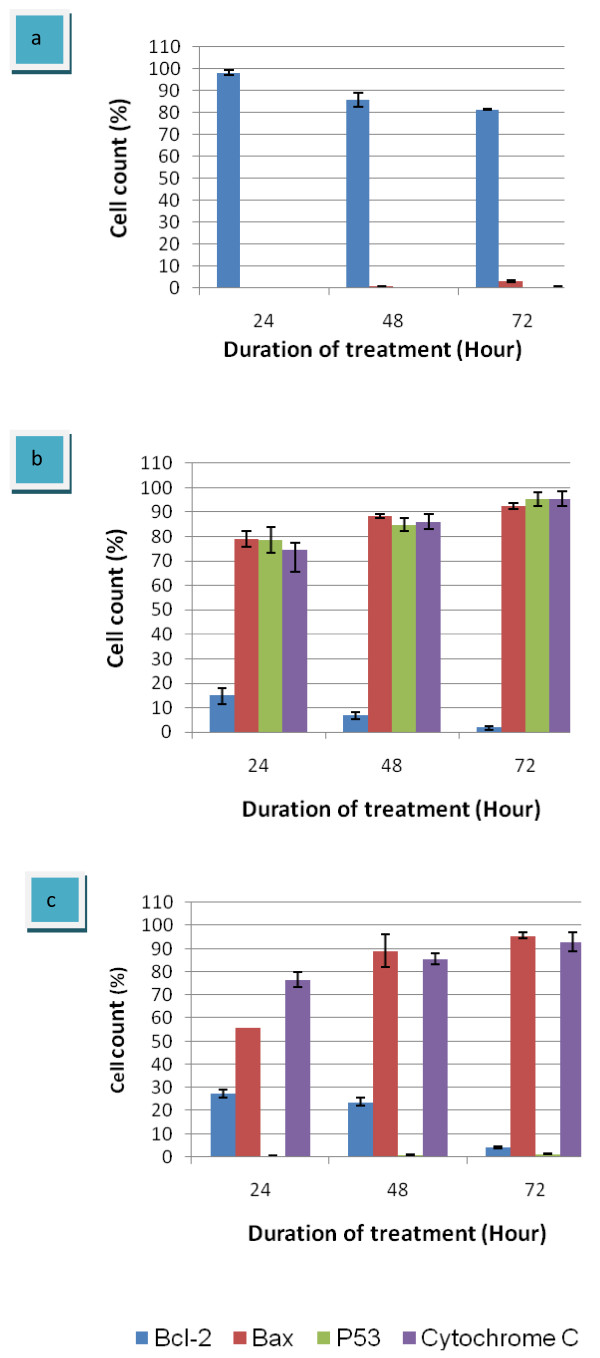
**Graphs summarized the expression levels of Bcl-2, Bax, p53 and Cytochrome C**. a = untreated HepG2 cells; b = HepG2 cells treated-eurycomanone; c = HepG2 cells treated-vinblastine sulfate. The values of the bars represented mean ± S.D. of three independent experiments. P < 0.05.

The analysis of the effect of eurycomanone on the expression of Bcl-2 protein in HepG2 cells using flow cytometry shows that eurycomanone can inhibit Bcl-2 activity as the time of exposure increases. The expression of Bcl-2 protein is lower as shown in figure 9b compared to the untreated HepG2 cells (figure 9a). In untreated HepG2, a great accumulation of Bcl-2 proteins were observed. At 24 hours of treatment duration, 98.3% of Bcl-2 was detected and no significant increases for the other proteins, Bax, p53 and cytochrome C. Following treatment at 48 h and 72 hours, the Bcl-2 levels slowly decreased to 86.0% and 81.5%, respectively (figure 10a). Furthermore, the expression of Bax protein have been seen to increase but only in a small value which is 0.7% at 48 hours to 3.1% at 72 hours.

HepG2 treated-eurycomanone inhibited the expression levels of Bcl-2. On the contrary from untreated cells, at 24 hours of exposure with eurycomanone, Bcl-2 levels dramatically decreased to 14.9% and again, at 48 hours, the levels decreased to 6.9% and only 1.8% after 72 hours of treatment. The accumulation of Bax proteins were significantly occurred (figure 10b). The percentage of 79.1% of Bax proteins were calculated at 24 hours of incubation with eurycomanone. The expression levels of Bax increases in a time dependent. At 48 hours, the Bax proteins increased to 88.5% and further increased to 92.3%. These results also supported by the previous studied which shows that eurycomanone suppressed the levels of Bcl-2 proteins and increased the expression levels of Bax in Hela cells (Nurkhasanah et al. 2008).

As for in HepG2 treated-vinblastine sulfate, Bcl-2 levels also inhibit in a time dependent manner. From 27.3% at 24 hours, the expression levels of Bcl-2 decreased to 23.7% and greatly reduced to 4.1% at 74 hours. The levels of Bax proteins also dramatically increased. The expression levels of 55.8% and 89.1% were detected at 24 and 48 hours, respectively. The expression levels showed a maximal value at 72 hours with percentage level of 95.6% (figure 10c).

p53 protein was detected to increase from 24 to 72 hours of exposure, thus recommended that eurycomanone induces apoptosis dependent p53 pathway. As shown in figure 9c, eurycomanone triggered strong p53 protein expression in HepG2 cells with 78.5% of p53 proteins levels were expressed at 24 hours of exposure and at 48 hours, the expression level increase to 84.9% and 95.3% at 72 hours. Along with decreasing of p53 levels, cytochrome C also increased in a time dependent manner (figure 9d). The percentage of 74.4% was determined at 24 hours of treatment and 86.1% at 48 hours. At 72 hours, the level of cytochrome C proteins showed a maximal value of 95.5%.

Furthermore, a different pattern of HepG2-treated vinblastine sulfate was observed in the level of p53 proteins and cytochrome C. As we can see from the figure 10c, there were no significant increased in a level of p53 in HepG2 cells treated-vinblastine sulfate for all treatment durations. These results show that, vinblastine sulfate enhances apoptosis in HepG2 cells via a p53-mediated pathway. However, the expression levels of cytochrome C were increased from 76.6% at 24 hours to 85.6% and 92.8% at 48 and 72 hours, respectively.

## Discussion

Plants have formed the basis of sophisticated traditional medicine systems among which are Ayuverdic, Yunani, Chinese amongst others. These systems of medicine have given rise to some important drugs still in use today [32]. Plants also are valuable source of new natural products. Despite the availability of different approaches for the discovery of therapeuticals, natural products still remain one of the best reservoirs of new molecules [33]. The search for new molecules, nowadays, has taken slightly different route where the science of ethnobotany and enthnopharmacognosy are being used as guide to lead the chemist towards different sources and classes of compounds. Natural products play an important role in chemotherapy, having contributed considerably to approximately 60 available cancer chemotherapeutic drugs [34]. The need to develop more effective antitumor drugs has prompted investigators to explore new sources of pharmacologically-active compounds, especially from natural products [35].

Eurycomanone found in *E. longifolia *is one of the novel compounds with promising potencies to be developed as a new chemotherapeutic agent. In this study, it was found that eurycomanone exerted anti-proliferative activity in HepG2 cells with an IC_50 _value of 3.8 ± 0.12 μg/ml. Previous study of eurycomanone on Hela cells and MCF-7 cells also showed the apoptotic effect [21,36]. This fact increases evidence that chemotherapeutic agents induce cancer cell death through the mechanism of apoptosis [37]. Additionally, cytotoxicity results show that eurycomanone gave an IC_50 _of 17 ± 0.15 μg/ml towards normal liver cells, Chang's Liver compared to tamoxifen with very low IC_50 _value (1.4 ± 0.31 μg/ml). It is showed that eurycomanone more selective than tamoxifen. This kind of cytoselectivity may reduce a side effect which commonly occurred during chemotherapy. This finding may lead to develop a new drug as an alternative medicine to treat liver cancer. The previous study also showed the minimum effect of eurycomanone on noncancerous MDBK and Vero cells [21] and also noncancerous breast cells (MCF-10A) [36].

Based on the result of Hoechst staining, it was found that treatment with eurycomanone induced DNA fragmentation in HepG2 cells. Internucleosomal DNA fragmentation is the primary biochemical characteristic to indicate an early event of apoptosis and it represents a point of no return from the path to cell death [38]. Shrinkage of cells, DNA condensations, nuclear and plasma membrane convulsion and nuclear fragmentation were all observed in HepG2 cells treated-eurycomanone. Positive control of tamoxifen-treated cells displayed similar nuclear fluorescence indicating that the data obtained can be accepted. These observations provide evidence that an apoptotic pathway is occurring with the eurycomanone treatment.

The term of 'apoptosis' was introduced into modern science by Kerr et al. in 1972 [26] to describe the special morphology of physiological cell death. The word itself is an ancient Greek word meaning the 'falling off' of leaves from a tree or petal from a flower and was originally used in medical and philosophical writings of classical Greek and Roman times. Apoptosis or programmed cell death represents the regulated activation of a pre-existing death program encoded genome [39,40]. Apoptosis has been shown as a significant way of cell death after cytotoxic drug treatment in a variety of cancer types. The induction of apoptosis has been recognized as a strategy for the identification of anticancer drugs [41]. There is substantial evidence that alteration in the cellular and molecular pathways that control the cell cycle and apoptosis may change the sensitivity and resistance to anticancer agents [42].

There is an increasing realization that chemotherapeutic agents act primarily by inducing cancer cell death through the mechanism of apoptosis [43]. In the present study, we provide evident eurycomanone is able to induce apoptosis in the human liver cancer cell line, HepG2. The morphology changes took place in HepG2 cells exposed to *E. longifolia *extract and eurycomanone. When stained with a Hoechst nuclear fluorochrome, the chromatin of the eurycomanone-treated HepG2 cells can be seen as condensed into lumps, thus exhibiting the punctuated morphology typical of apoptotic cells. The ability of eurycomanone to inhibit cancer cells proliferation and induce apoptosis further purports it as a potential anticancer agent in liver carcinomas, thus making it a promising agent for chemotherapy, which merits further study.

The amount of apoptotic cells was calculated based on the appearance of cells in G0. G0 phase is corresponds to apoptotic cells. Although there was an increase of apoptosis in eurycomanone-treated cells, the magnitude of change was relatively small in comparison to the growth inhibition. Distribution of the G2/M phase cells in the population increase while the percent of apoptotic cells rose from 3.5% to 10.4%. This is also evident that the G2/M phase became the dominant phase in cells treated with 5 μg/ml of eurycomanone in a time-dependent manner with a late apoptosis. These finding indicate that at the ranges of concentration studied, the antiproliferative effect of eurycomanone on HepG2 cells could be attributed primarily to the induction of G2/M arrest, with less contribution of cell division rather than DNA synthesis.

A flow cytometry results for the DNA content showed HepG2 cells treated with eurycomanone increased at G2/M phase compared to the untreated. It was suggested that, eurycomanone appears to affect processes that are could inhibit the cell proliferation by inducing G2/M arrest in a time-dependent manner in HepG2 cells. The G2/M arrest in HepG2 cells could be induced through up-regulation or induction of p53. A few researchs have been done towards the action of several natural products and chemical against HepG2 cells. Ho and collegues, 2007 [44], reported that Taiwanin, a lignin isolated from *Taiwania cryptomerioides *Hayata, induced cell cycle arrest of HepG2 cells at G2/M phase. Apigenin, a common dietary flavanoid abundantly present in fruits and vegetables also reported to induce cell cycle arrest of HepG2 cells at G2/M phase through p53 dependent pathway [31]. Besides, Shieh et al. 2003 [45] reported that emodin or 1,3,8-trihydroxy-6-methyl-anthraquinone displays effective inhibitory effects on the growth of various human hepatoma cell lines and stimulates the expression of p53 and p21 that resulted in the cell cycle arrest of HepG2/C3A cells at G2/M phase. The known members of inhibitor of growth (ING) gene family are considered as candidate tumor suppressor genes, ING4, a novel member of ING family, is reported to negatively regulate the cell growth with significant G2/M arrest of cell cycle in HepG2 cells [46].

The studies of the effect of eurycomanone on the expression of Bcl-2 protein in HepG2 cells shows that eurycomanone can inhibit Bcl-2 activity, as the time of exposure increases, the expression of Bcl-2 protein is lowered. Bcl-2 is an anti-apoptotis gene that indirectly inhibits apoptosis by inhibiting the opening of permeability transition (PT) channels on the mitochondrian and hence the lowering of mitochondrial potential [47,48], and by blocking the release of cytochrome C from mitochondrion. As a conclusion from the finding, HepG2 cells are relatively sensitive to the cytotoxic effect of eurycomanone which induces apoptosis in HepG2 cells by inhibiting the Bcl-2 protein. The level of Bax was increased and concomitantly that of Bcl-2 was decreased, suggesting that eurycomanone induced apoptosis through up-regulation of Bax and down-regulation of Bcl-2 level.

Alterations of the p53 gene are the most frequent genetic change in human cancers. It is estimated that about 50% of all human malignancies contain mutations of this gene [49]. As p53 has been reported to play the role of transcriptional activator, active p53 induces the transcription of many genes, including many apoptosis-related genes. The Bax gene has recently been found to be a transcriptional target of p53 and could be up-regulated in response to a variety of p53-dependent apoptosis triggers [50]. This present study demonstrates that the up-regulation of Bax is due to the activated p53. The data also suggest that the inhibition of cell cycle progression might be associated with level alterations of the cell cycle related p53. This finding also suggests that the elevation of p53 expression may play an important role in eurycomanone induced G2/M arrest and apoptosis in HepG2 cells. Previous studies also showed that p53 and p21 are necessary to maintain a G2/M arrest and apoptosis following DNA damage [31].

The activation and action of p53 is believed to be a pivotal step in the mechanism of apoptosis [51]. Nevertheless, the effect of p53 on the sensitivity of HepG2 cells to eurycomanone has not been as extensively studied. Interestingly, from this study, we found that a high level of p53 protein accumulated in the cytoplasm of HepG2 cells. Many studies have suggested that this protein is a nuclear protein. There may be a possibility that may cause the cytoplasmic accumulation of p53. We believed that p53 may act on other pro-apoptotic protiens to modulate their functions in cytoplasm. p53 may dictate the translocation of Bax protein from the cytoplasm into mitochondria, with consequent cytochrome C release [52].

Recent studies show that cytochrome C participates in activating the programme of cell death. Addition of exogenous cytochrome C to cytosol can trigger apoptotic programmes in a cell-free apoptosis system [53,54], and microinjection of cytochrome C to cytosol also results in induction of apoptosis [55]. Since Bax has been seen shown to be a downstream mediator of p53, it is reasonable to hypothesize that cytochrome C may be involved in P53-induced apoptosis. As we can see from this experiment, the expression levels of Bax and cytochrome C were increases, which was supported this hypothesis. In addition, Bcl-2 may regulate apoptosis by controlling cytochrome C release. Release of cytochrome C in cells undergoing apoptosis could be prevented by overexpression of Bcl-2. The inhibitory effect of eurycomanone on Bcl-2 ultimately leads to an increase in the content of cytochrome C, which directly activates the cascade reaction of caspase and enhances apoptosis. Increased levels of Bax protein have been shown to be able to directly induce release of cytochrome C conceivably by forming a pore in the outer membrane of mitochondria that allow cytochrome C to leak out [56].

In conclusion, we suggest that, eurycomanone inhibit cells growth at G2/M phase and prevent cells from undergo mitosis process. The mechanism of cell death induce-eurycomanone was via apoptosis. Eurycomanone enhances apoptosis in HepG2 cell in a p53-dependent pathway. The large number of p53 expression induces translocation of Bax from cytoplasm into mitochondrial which lead to cytochrome C release and induces apoptosis process to occur. Despite the finding of p53 involvement in eurycomanone cytotoxicity, the exact downstream target of p53 is unclear, which require further investigation. However, we suggest that induction of p53-mediated apoptosis is, at least in part, a possible explanation for the anti-liver-carcinogenic effect of eurycomanone, and that eurycomanone has a potential to prevent cancer.

## Abbreviations

FDA: Food and Drug Administration; EBV: Epstein-Barr virus; HepG2 cells: human liver cancer cells; HM3KO cells: human skin cancer cells; Hela cells: human cervical cancer cells; CaOV3 cells: human ovarian cancer cells; CCD11114sk cells: human fibroblast cells; BuOH: butanol; TLC: thin layer chromatography; HPLC: high performance liquid chromatography; MTT: 3-(4,5-Dimethylthiazol-2-yl)-2,5-diphenyltetrazolium bromide; ELISA: Enzyme Linked Immuno Sorbent Assay; Et_2_O: diethylether; Et_O_Ac: ethyl acetate; EtOH: ethanol; H_2_O: water; ATCC: American Type Culture Collection; DMEM: Dulbecco's Modified Eagle's Medium; EDTA: ethylenediaminetetraacetic acid; DMSO: dimethylsulfoxide; OD: optical density; DNA: deoxyribonucleic acid; IC_50_: inhibition concentration to kill 50% of cells population; LD_50_: lethal dose to kill 50% of animals population.

## Competing interests

Before this, there are other researchers working on this active compound, eurycomanone. However they are working towards other cancer cells but not liver cancer cell. Furthermore, the research has been conducted for 2 years ago.

## Authors' contributions

YZ carried out the whole research and drafted the manuscript. All authors read and approved the final manuscript.
